# Management of increased posterior tibial slope in ACL deficiency: A narrative review and recommendation of the Osteotomy Committee of the German Knee Society

**DOI:** 10.1002/ksa.70549

**Published:** 2026-08-03

**Authors:** Philipp Mayer, Julian Fürmetz, Gerrit Bode, Peter Angele, Marc‐Daniel Ahrend, Sebastian Leutheuser, Christoph Lutter, Svea Faber, Maximilian Michael Mueller, Jörg Harrer, Felix Ferner, Björn Vogt, Florian B. Imhoff, Philipp Schuster

**Affiliations:** ^1^ Orthopedic Hospital Markgroeningen Centre for Sports Orthopedics and Special Joint Surgery Markgroeningen Germany; ^2^ Osteotomy Committee of the German Knee Society (Deutsche Kniegesellschaft) Schwarzenbeck Germany; ^3^ Department of Sports Traumatology and Arthroscopic Surgery BG Klinikum Murnau Murnau Germany; ^4^ Department of Orthopedics and Trauma Surgery Musculoskeletal University Center Munich (MUM), University Hospital, LMU Munich Munich Germany; ^5^ Praxisklinik2000 Freiburg Germany; ^6^ Department of Orthopedics and Trauma Surgery Freiburg University Hospital Freiburg Germany; ^7^ Clinic for Trauma and Reconstructive Surgery University Hospital Regensburg Regensburg Germany; ^8^ Sporthopaedicum Regensburg Regensburg Germany; ^9^ Department of Traumatology and Reconstructive Surgery, BG Trauma Center Tübingen Eberhard Karls University Tübingen Tübingen Germany; ^10^ Department of Orthopedic Surgery and Sports Traumatology, Sana Medical Centre Witten/Herdecke University Cologne Germany; ^11^ Department of Orthopaedics Rostock University Medical Center Rostock Germany; ^12^ Department of Trauma Surgery Orthopaedics and Sports Traumatology, BG Klinikum Hamburg Hamburg Germany; ^13^ Department of Orthopaedic Surgery, Hospital for Special Surgery, NewYork‐Presbyterian Weill Medical College of Cornell University New York New York USA; ^14^ Department of Orthopedics and Traumatology Sana Klinikum Lichtenfels Germany; ^15^ Pediatric Orthopedics, Deformity Reconstruction and Foot Surgery Muenster University Hospital Muenster Germany; ^16^ General Orthopedics and Tumor Orthopedics Muenster University Hospital Muenster Germany; ^17^ Department of Orthopaedics and Traumatology University Hospital of Basel Basel Switzerland; ^18^ Department of Orthopedics and Traumatology, Clinic Nuremberg Paracelsus Medical University Nuremberg Germany

**Keywords:** ACL insufficiency, ACL revision, ACL rupture, posterior tibial slope, slope osteotomy

## Abstract

Increased posterior tibial slope (PTS) is a known risk factor for anterior cruciate ligament (ACL) deficiency and graft failure after ACL reconstruction (ACLR). Although many studies investigated different aspects related to the PTS, there is still a lack of comprehensive recommendations and practical guidelines, especially with regard to radiographic analysis, surgical indication and surgical technique. The aim of this narrative review is to present such recommendations, based on the existing literature (evidence Level 3–4), supplemented by the expert opinions of the Osteotomy Committee of the German Knee Society. A review of the literature was conducted to summarize the current evidence concerning biomechanical background, radiological measurement, indication for surgical correction and surgical techniques. Results were presented to a panel of surgeons with recognized expertise in the treatment of ACL injuries and wide experience in surgical correction of the PTS, and consensus was formed by informal discussion. These recommendations were developed and included: (1) measuring the PTS should be performed on long lateral radiographs using the mechanical axis, with attention to a correct rotational alignment (exact superimposition of the posterior tibial condyles). (2) Indication for surgical correction does not depend on a single fixed cut‐off value of the PTS. It should be based on an individual risk assessment (e.g., degree of anterior tibial translation and instability, meniscal integrity, number and quality of previous ACLR, trauma mechanism: contact vs. non‐contact, contralateral injury). (3) For correction of pathologic PTS, different surgical techniques of anterior tibial closing wedge osteotomies have been reported without clear superiority of one technique. However, an infratuberositary approach is recommended, because it has been shown to be technically easier and safer.

**Level of Evidence:**

Level V, narrative review and expert opinion.

**Abbreviations**
AA: anatomical axis
ACL: anterior cruciate ligament
ACLR: anterior cruciate ligament reconstruction
ACW: anterior tibial closing wedge osteotomy
ATT: anterior tibial translation
FU: follow‐up
IT: infratuberositary
MA: mechanical axis
mm: millimetre
PAA: proximal anatomical axis
PROMs: patient‐reported outcome measures
PTS: posterior tibial slope
SATT: static anterior tibial translation
SSD: side‐to‐side difference
ST: supratuberositary
TT: transtuberositary

## INTRODUCTION

The management of increased posterior tibial slope (PTS) in anterior cruciate ligament (ACL) deficiency is a highly discussed aspect in the field of ACL reconstructive surgery, particularly in the context of ACL reconstruction (ACLR) failure. Increased PTS has been identified as a significant risk factor for both primary ACL injuries and failure following ACLR [14, 68, 87, 111]. In the literature, the individual expression of the PTS shows relevant differences between ethnic groups [6, 7, 74]. Further, it was reported that paediatric tibial tubercle fractures or Morbus Osgood–Schlatter disease are related to increased values of PTS [30, 66, 97].

Nevertheless, despite the known impact of increased PTS on ACL‐ or ACL graft deficiency, it remains difficult to extract clear treatment indications from the current literature [78, 95, 111]. Concerning the measurement of increased PTS, a variety of methods have been published [40, 46, 104]. Each method has its own advantages and disadvantages, and individual physiological values. Initially, the measurement on short radiographs was common. Along with advances and validation in measurement methods, the use of long lateral radiographs has become more widespread. Further, there is an ongoing debate about clear criteria for evidence‐based recommendations at what extent of the PTS a correction osteotomy is recommended to achieve a solid reduction of the failure risk after ACLR and which concomitant risk factors are likely to influence or even multiply this risk status [23, 103]. While a growing body of literature addresses these questions, including systematic reviews and an international Delphi consensus, there remains a lack of high‐level comparative evidence (Level 1–2), and no universally accepted standard on all aspects of PTS management has been established [107].

This article provides a narrative review of the current literature and clinical implications of increased PTS in the setting of ACL deficiency. Further, technical aspects of various surgical interventions in managing this condition are outlined and a recommendation of the Osteotomy Committee of the German Knee Society for the management of patients with increased PTS in case of ACL deficiency is given.

## METHODS

A group of 14 specialized reconstructive knee surgeons, all members of the Osteotomy Committee of the German Knee Society, with high expertise in osteotomies and the treatment of ACL injuries, built the group discussing consensus recommendations. The fields of interest were defined by a scientific organization team (P. M. and P. S.) who moderated the discussion. As fields of interest were defined: biomechanical background, radiographic assessment of PTS, indication for PTS decreasing osteotomy and surgical technique of anterior closing wedge (ACW) slope decreasing osteotomies. A database search was performed using PubMed and EMBASE, searching for the topics named above; no date range was applied; all levels of evidence (I–V) were included in the search, searching for original research papers, systematic reviews and meta‐analyses. The selected publications formed the basis for the narrative review. The defined topics were then discussed in the expert group and, based on the available data and results of the discussion, the best possible treatment recommendations were developed by informal discussion. Due to the character of the study, no study approval was required.

### Biomechanical background

Already in 1977, Williams and Lissner and later Nordin and Frank analysed forces acting on the extended knee in the sagittal plane. By differentiating between compressive and shear components, it was demonstrated that shear forces promoted an anterior translation of the tibia relative to the femur [54, 69].

In 1997, a mathematical model was introduced to evaluate the effects of tibial surface geometry and ligament orientation on cruciate ligament loading during simulated knee loading. It was demonstrated that an increased posterior tilt of the tibial plateau, representing the PTS, augmented loading of the ACL, emphasizing the critical role of osseous morphology in knee stability [44].

Subsequently, Liu et al. investigated the effects of anthropometric and mechanical variations on functional instability in ACL‐deficient knees using a mathematical knee model. They identified the PTS as a major determinant of anterior tibial translation (ATT), with steeper slopes resulting in increased instability and reduced effectiveness of dynamic hamstring compensation. Specifically, a PTS of 12° resulted in 15.2 mm of ATT, whereas a PTS of 4° produced only 9.1 mm [55]. These observations were later confirmed experimentally in a cadaveric robotic study investigating the biomechanical consequences of increasing PTS. The authors demonstrated that greater PTS caused anterior displacement of the tibial resting position, which became further accentuated under axial compressive loading [28].

Further evidence was provided by Bernhardson et al., who reported a linear increase in ACL graft forces with increasing PTS during axial loading, independent of knee flexion angle, with maximal graft loading occurring in full extension [4]. Similarly, Marouane et al. used a combined musculoskeletal and finite element knee model to analyse the influence of PTS during gait. Increasing PTS resulted in substantially greater ATT and ACL loading, whereas slope reduction significantly decreased forces acting on the ACL, suggesting a potential protective biomechanical effect [58].

Other investigations examined the impact of increasing PTS on joint kinematics and contact mechanics in both intact and ACL‐deficient knees during the gait cycle. In their finite element‐musculoskeletal framework model, a PTS angle greater than 11.4° led to significant knee instability, greater posterior femoral translation and increased external rotation, particularly in ACL‐deficient knees. In addition, elevated contact pressures within the medial meniscus and tibial cartilage were observed, potentially contributing to secondary meniscal injury and degenerative joint changes [98]. Furthermore, it was reported that increased lateral PTS and greater lateral‐to‐medial slope asymmetry correlated significantly with increased tibial acceleration during the pivot‐shift test, indicating enhanced anterolateral rotational laxity [49].

Several subsequent investigations evaluated the biomechanical consequences of PTS‐reducing osteotomies. In 2019, Imhoff et al. demonstrated that slope‐reducing tibial osteotomy significantly decreased ACL graft forces under axial compression and reduced ATT in ACL‐deficient knees [43]. In a follow‐up (FU) study, the same group assessed the combined effects of varus correction and slope correction using medial high tibial osteotomy. Isolated varus correction increased ATT, whereas isolated slope correction significantly reduced ATT. The greatest biomechanical benefit was observed when both corrections were combined, resulting in substantial reductions in ACL graft forces under axial loading [42].

More recently, Pearce et al. analysed the biomechanical influence of PTS variation on ACLR with and without additional lateral extra‐articular tenodesis (LET). Increasing PTS produced a linear increase in ACL graft loading, whereas LET reduced graft forces by 8.3% at 30° of knee flexion compared with isolated ACLR. However, slope reduction demonstrated an even greater biomechanical effect, decreasing graft forces by 17%–22% per 5° correction and achieving an overall reduction of 46% by reducing PTS from 20° to 5° [78].

### Radiographic assessment of PTS

In the past, multiple techniques for measuring the PTS were published. Most of these techniques used lateral radiographs [25, 89, 117]. Measurement methods for computed tomography (CT) and magnetic resonance imaging (MRI) were published [40, 104]. Due to this variety of methods to analyse the PTS, it remains difficult to compare the published results, as every measurement method has its own range of physiological values [39, 89, 115, 117]. In daily clinical routine, the measurement on lateral radiographs remains the current standard. The methods to measure the PTS on lateral radiographs can be divided into two groups, one using only the proximal part of the tibia to determine the PTS and the other using full‐length lateral radiographs of the tibia. Overall, techniques based on long lateral radiographs are capable of identifying posterior tibial slope alterations originating from distal alignment changes [37]. Similarly, when planning correction along the frontal axis, determining the mechanical axis (MA) based on long leg X‐rays is also considered the generally accepted standard.

Most common techniques to analyse PTS on radiographs are described below and displayed in Figure 1. Intra‐ and interobserver reliability is high for the presented measurement techniques [40, 45, 111, 114]. Analysis of PTS on MRI or CT scans is not included in the further review, since they are not used as standard in everyday clinical practice.

**Figure 1:**
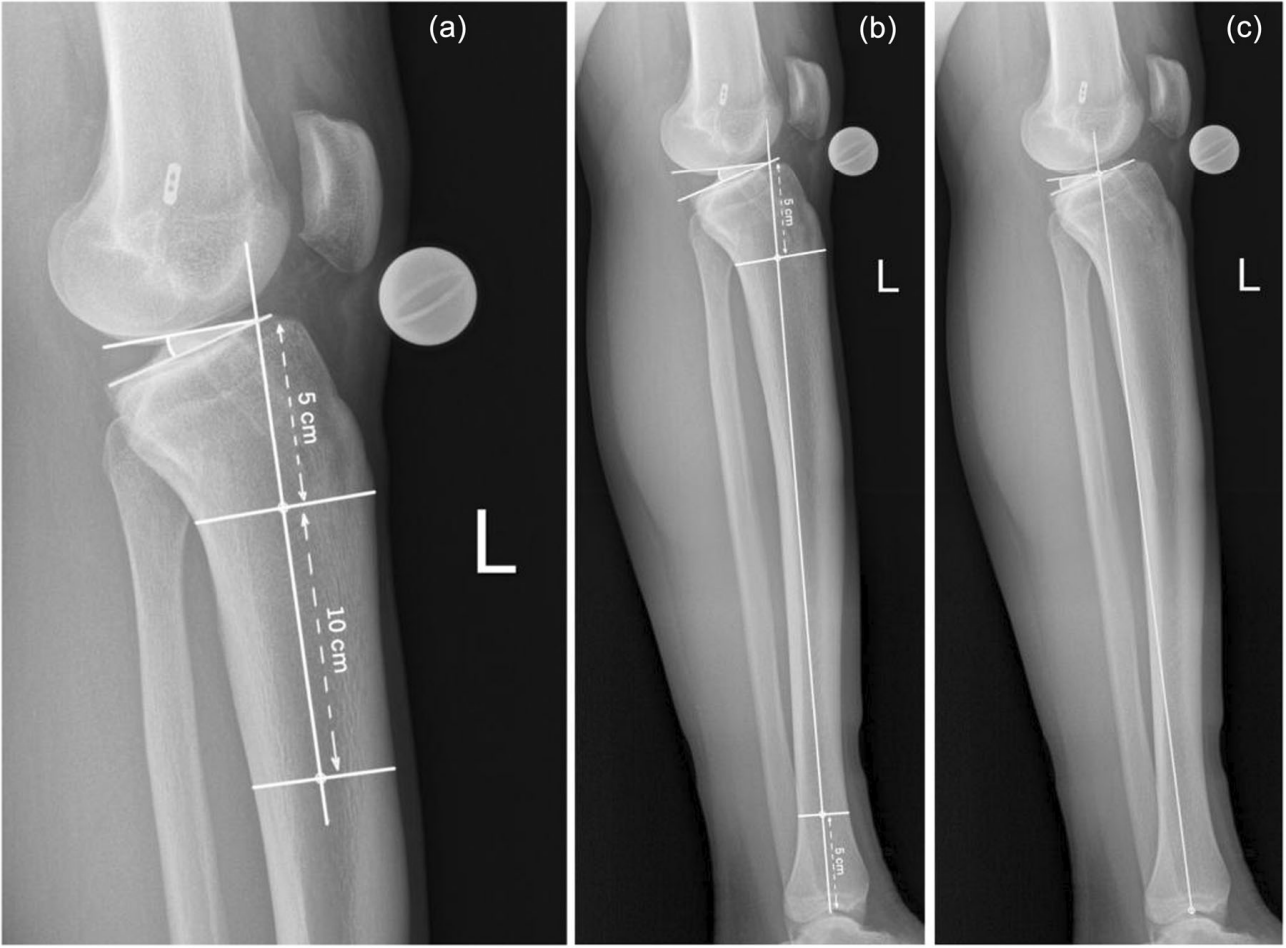
Measurement of posterior tibial slope (PTS) with the (a) proximal anatomical axis (PAA) according to Yoo et al. [117]; (b) anatomical axis (AA) as described by Dean et al. [13]; (c) mechanical axis (MA) as described by Yoo et al. [117].

### Proximal anatomical axis (PAA)

The measurement with the PAA was first described by Dejour and Bonnin in 1994 [21]. In this method, the first diaphyseal reference should be located just below the tibial tubercle, and the second reference line should be located 10 cm distal to the first reference line on the lateral radiograph. The PAA is defined by a line connecting the midpoints of the two reference lines described above. This technique was modified by Yoo et al. later in 2008 [117]. The PAA, as described by Yoo et al., is defined as a line connecting the midpoint of the tibial shaft 5 and 15 cm below the joint line; the angle between a tangential line to the medial tibial plateau and a line orthogonal to the PAA expresses the PTS. These techniques for PTS measurement only require the proximal part of the tibia being displayed on lateral radiographs, hence allowing the measurement of the PTS on standard short lateral radiographs. This technique is most commonly used in publications discussing ACLR failure and often, values exceeding 12° of PTS are stated to show increased risks for ACL deficiency [87, 103, 111].

### Anatomical axis (AA)

The measurement of the PTS with the AA is performed on long lateral radiographs. As initially described by Paley et al., it is measured between the joint line in the sagittal plane and the mid‐diaphyseal line of the lateral tibia [73]. Dean et al. described a measurement method adapted to the technique described before. In their publication, they used two midpoints of the tibial shaft, one 5 cm below the knee joint, another 5 cm above the ankle joint. These two points establish the AA in the sagittal plane [13]. The angle between a tangential line to the medial tibial plateau and a line orthogonal to the AA expresses the PTS. With respect to ACL deficiency, no clear cut‐off values are published.

### Mechanical axis (MA)

First described by Yoshioka and Cooke in 1989 and then adapted by Yoo et al. in 2008, the measurement of the PTS with the MA is performed on long lateral radiographs, too [117, 118]. As defined by Yoo et al., the MA of the tibia in a sagittal plane is measured as a line connecting the midpoint of the medial tibial plateau and the midpoint of the tibial plafond. The angle between a tangential line to the medial tibial plateau and a line orthogonal to the MA expresses the PTS. For the MA, no clear cut‐off values for increased risk of ACL deficiency are described.

### Differences between techniques for PTS measurement

Several publications investigated the differences between the reference axis described above. Comparing all three methods for radiologic PTS analysis, Dean et al. reported significantly increased values for the PTS, measured using an anatomical axis (PAA or AA), compared to the measurement using the MA. When comparing the PAA and the AA, there was no significant difference reported [13]. In general, there is a mean difference between the MA and PAA of about 1.2°, with the PAA showing higher values [13, 25, 37, 60, 117]. Under closer inspection, the differences described between the MA and PAA do show interindividual differences [60, 79]. Due to a tibial bowing, Hees et al. reported on the phenomenon of underestimating the PTS with the PAA compared to the MA. This resulted from a bowing deformity of the tibia in the sagittal plane, which was located distally, not covered by the measurement of the PAA [37]. Further, it has to be considered that there are outliers regarding the general assumption that PTS values of the PAA are always higher than those of the MA. More detailed reports indicate that the differences between the MA and the PAA are inconsistent [60, 79]. In an analysis of 196 long lateral radiographs and a comparison of the MA an PAA, it was demonstrated that, contrary to the usual expectation, in 16% the PTS measured with the PAA showed smaller PTS values than measured with the MA. Especially in the area between 9° and 14° of PTS (measured with the MA), the proportion of outliers, as mentioned above, increased to 20% [60]. It was concluded that the PAA cannot be recommended as a screening method, as it is not possible to predict the individual difference between the MA and the PAA unless both PTS values were measured [60]. By referencing the MA across the entire lateral tibia on long lateral radiographs, distally induced malalignment can also be identified. In contrast, distally induced sagittal alignment changes cannot be detected on short lateral radiographs when using the PAA.

### Quality criteria for measurement of PTS on lateral radiographs

Multiple publications reported a change in PTS measurement due to rotational malalignment of the tibia on lateral radiographs. The findings of these publications are in line, unanimously recommending avoiding rotational malalignment [51, 60, 79, 105, 114]. In a sawbone study, tibial external rotation led to an underestimation of the PTS, whereas tibial internal rotation resulted in increased PTS values due to projection errors [61]. It is still under discussion which parameters detect rotational malalignment adequately. Some authors proposed the posterior femoral condyles as a landmark to detect rotational malalignment on lateral radiographs [33]. When the overlap of the femoral condyles was ≥5 mm, the probability that the difference between the two raters' measurements exceeded 2° increased significantly. As shown in another recent publication, malrotation can even occur within the knee joint itself [41]. A broad variation of the individual knee version could be shown in their publication. Therefore, it must be concluded that a correct alignment of the posterior femoral condyles does not automatically imply a correct rotational alignment of the posterior tibial condyles in the sagittal plane (Figure 2). Weinberg et al., as well as Mayer et al., suggested using the posterior tibial condyles as a reference to judge the rotational alignment on lateral tibial radiographs, aiming for an exact superimposition of the medial and lateral posterior tibial condyles as a sign of neutral radiographic rotation [60, 61, 114]. In the opinion of the authors, it is certainly reasonable to measure the tibial slope on a X‐ray aligned with the tibia, as it is the identical positioning in which the surgical correction is performed. However, it should be noted that, depending on the individual torsional alignment, this projection of the sagittal tibia may not necessarily be perpendicular to the frontal axis. Consequently, a correction in this projection plane does not fully correspond to a strict sagittal correction. Subsequently, changes in frontal plane alignment can occur [48, 109]. Still, as mentioned above, this remains the positioning in which the osteotomy can be performed with the highest precision, if the goal is to establish a stable hinge axis parallel to the posterior tibia. From a geometrical point of view, when aligned with the femoral condyles, the tendency for frontal plane changes might be slightly lower. However, unintentional frontal plane changes still cannot be excluded, and this positioning is associated with the described disadvantage of overall reduced accuracy and the fact that this ultimately tends to result in a more unstable hinge point rather than a more stable hinge axis due to angulation and rotation of the osteotomy plane in relation to the tibia.

**Figure 2:**
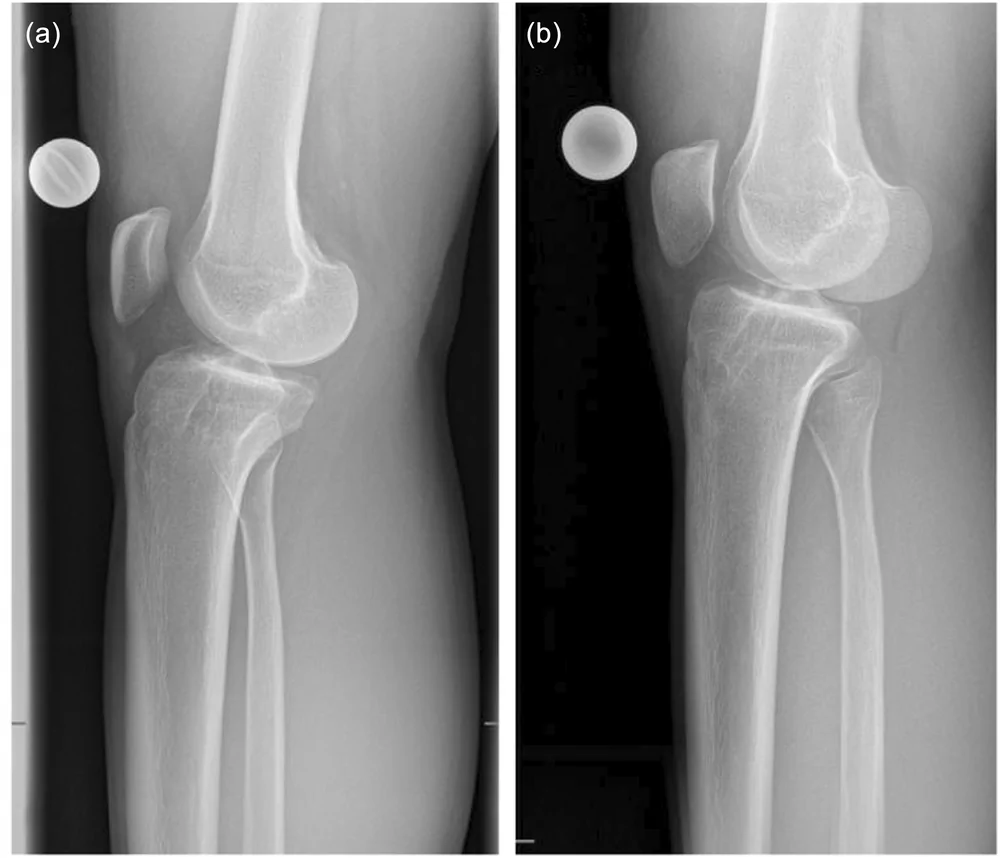
Radiographs of a case with increased knee version; both X‐rays show the same patient: (a) femoral condyles are superimposed exactly, tibial malrotation occurs; (b) tibial condyles are superimposed exactly, femoral malrotation occurs.

### Measurement of static anterior tibial translation (SATT) (Figure 3)

**Figure 3:**
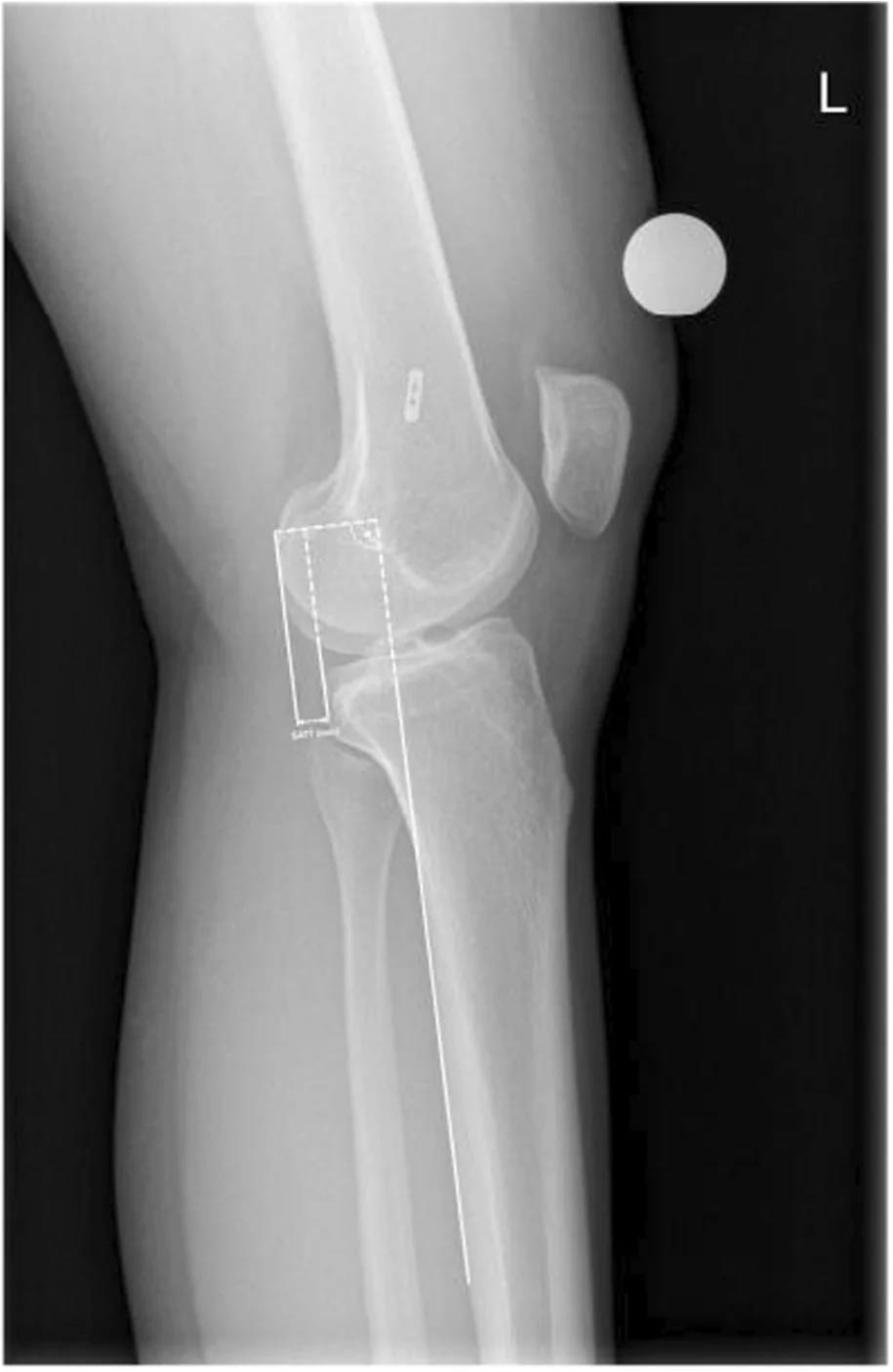
measurement of the static anterior tibial translation (SATT) on lateral radiographs; SATT is measured as the distance between the posterior femoral and tibial condyles parallel to the posterior tibial cortex [21].

Initially described by David Dejour and Bonnin in 1994, the SATT represents a relevant parameter in the context of increased PTS [16, 17, 21]. Measurement is performed using a reference line drawn along the posterior tibial cortex and can be obtained on both short lateral radiographs and full‐length radiographs. SATT is measured in millimetres (mm) using two lines parallel to the reference line: one tangential to the posterior border of the medial tibial plateau and the other tangential to the posterior femoral condyles. Radiographs are obtained in the standing position under axial load with the knee in slight flexion (20°–30°) [17, 107]. In particular, increased SATT values have been observed in patients with ACL deficiency and increased PTS [17]. For values exceeding 5 mm, combined with increased PTS (above 12°), higher rates for ACLR graft rupture are reported [64]. Clinical data suggest that isolated ACLR, even when combined with an LET, may not sufficiently compensate for the functional instability, and therefore may not result in a sustained reduction of SATT in the setting of increased PTS [80, 81]. Accordingly, SATT may be considered a parameter reflecting functional ACL insufficiency. Anterior closing‐wedge (ACW) osteotomy with subsequent reduction of the PTS has been shown to achieve a decrease in SATT [12].

However, the aforementioned limitations associated with the assessment of these parameters on lateral radiographs must be considered. Measurement of SATT is likewise susceptible to inaccuracies resulting from improper rotational positioning on lateral radiographs, as the spatial relationship of the referenced anatomical landmarks changes with malrotation. Therefore, in the authors' opinion, accurate rotational positioning with precise superimposition of the posterior femoral and tibial condyles is essential when assessing SATT.

### Recommendation for measuring the PTS on lateral radiographs

– The PTS should ideally be assessed on long lateral radiographs of the tibia, as this enables additionally to identify distal alignment changes.
– The measurement using the MA is recommended as it offers the best estimation of the sagittal conditions under load.
– Rotational malpositioning or angulation of the tibia must be avoided, as this results in measurement inaccuracy on lateral radiographs due to projection errors. Therefore, the posterior medial and lateral tibial condyles should be superimposed exactly with a free projection of the joint line, as the same sort of precise sagittal view is required in the operating room for anterior tibial closing wedge osteotomy (ACW).
– Further, X‐rays should be taken in a standing position for measurement of the SATT under a loaded condition.
– When planning with wedge height in mm, an X‐ray reference sphere should be used for calibration and osteotomy planning.
– An automatic capturing and stitching technique, as common in full leg X‐rays, can be used to avoid projection‐related errors.

### Indication for PTS decreasing osteotomy

Regarding indications for slope correction, it must be distinguished if the problem is purely in the sagittal plane or whether an osteotomy in the frontal plane may also be indicated for other reasons—particularly in the case of pre‐existing monocompartimental osteoarthritic changes or pronounced peripheral instabilities (e.g., a varus thrust phenomenon) [102]. Corrections in the frontal plane can be combined with PTS corrections or additional ligament reconstruction(s) [10, 63, 85, 90, 102]. Overall, the surgical strategy must ultimately be considered on a highly individual basis depending on individual symptoms and circumstances: in general, the PTS can be corrected to an extent of about 7°–8° via a medially opening tibial osteotomy, but not to the same extent as if addressed directly from anterior [113]. With ACW osteotomies, however, it must be considered that the osteotomy may result in slight (additional) varization of around 1°, and deliberate changes in frontal axis using an ACW approach are very limited [63, 113]. Therefore, cases with high PTS and slight but relevant frontal plane deformity remain challenging. If both frontal and sagittal deformities are of crucial relevance, both should be adequately addressed. Adapted surgical strategies or the use of double‐level corrections might offer solutions in these cases [35, 67, 91].

The following recommendations refer to isolated corrections of PTS using ACW techniques without consideration of deformities in the frontal plane.

The criteria for indicating a PTS‐changing osteotomy evolved over time: the first two studies on PTS‐decreasing osteotomies published in 2014 and 2015 reported on five and nine cases of second revision ACLR with concomitant ACW osteotomy [19, 101]. Both studies reported excellent results, and both indicated osteotomy in a PTS > 12°. However, Dejour et al. used the PAA as reference and Sonnery‐Cottet et al. used the MA on a long radiograph for measurement of the PTS, respectively. In subsequent studies, further inclusion criteria were added, and also cases with first revision ACL were included: Akoto et al. reported on 20 cases of first revision ACLRs having a PTS ≥ 12° (according to the PAA), but a further inclusion criterion was a distinct anterior knee laxity with a side‐to‐side difference of more than 6 mm [2]. Dejour et al. also reported on 15 cases of first revision ACL together with osteotomy: [18] they used different thresholds of PTS according to the PAA depending on the meniscus integrity and the presence of an excessive ATT. With poor meniscus integrity and with excessive ATT, the threshold for additional osteotomy in their patients decreased from ≥10° (according to the PAA) to ≥8°. In 2022, Weiler et al reported on 76 slope‐decreasing osteotomies (58 in ACW technique) in various indications, from primary ACL injuries to fourth revision ACLR [113]. They proposed that the patients' activity level and the degree of anterior laxity should be analysed together with the PTS and previous interventions.

In 2023, Mabrouk et al. reported on 64 cases, using comparable inclusion criteria to those in the first descriptions 10 years before: second revision ACL and >12° of PTS [57]. However, they measured the PTS using the lateral plateau and the AA of the tibia. There is one study group reporting results of combined osteotomy and ACLR in the setting of primary ACL injuries. Their indication was a high PTS of >13° according to the MA and the presence of four further factors: chronic ACL insufficiency (>6 months), non‐contact injury, excessive ATT in extension (>10 mm) and medial meniscus posterior horn lesion [99, 108].

With regard to these published indication criteria, two essential points must be noted: First, although most of the reported clinical studies refer to a threshold of 12°, there are substantial differences in the actual indications, as the axis and measurement methods used to determine the PTS varied considerably between studies (Table 1) [18, 57, 86, 99, 101]. Therefore, comparability is very limited. Second, patient‐individual factors and conditions have become increasingly important in determining the indication over the last few years.

**Table 1:** Overview of clinical studies on anterior closed wedge osteotomies with regard to technique, indication and results.

Author	Year	Cases in FU	LoE	Technique	Indication	Aim of correction	Most important clinical results
Akoto et al. [2]	2020	20		TT + two‐stage	First revision + high‐grade anterior knee laxity (>6 mm SSD) ≥ 12° PAA	8°−10° PAA	After 30.5 ± 9.3 months: No graft failure SSD 7.2–1.1 mm (KT‐1000) No pivot in any case Good to excellent PROMs One complication (haematoma)
Dejour et al. [18]	2023	15		ST + single‐stage	First revision ≥10° PAA or ≥8° PAA + excessive anterior translation and poor meniscus status	0°−5° PAA	After 4.4 ± 1.5 years: no graft failure, no signs of osteoarthritis, good to excellent PROMs, one re‐operation (re‐tear of meniscus)
Dejour et al. [19] and Rozinthe et al. [86]	2015	9	4/3	ST + single‐stage	Second revision >12° PAA	3°−5° PAA	After 9.9 ± 3.0 years: No graft failure Good to excellent PROMs No complications
Mabrouk et al. [57]	2023	64		ST + single‐stage	≥2nd revision >12° AA (lateral tibial plateau measured)	4°−6° (AA with lateral tibial plateau measured)	After 3.0 ± 0.8 years: Three failures (4.7%) Good to excellent PROMs (not preinjury level) 50% Return‐to‐impact‐sports 25% Return to preinjury sports level Recurvatum in one‐third, but all Asymptomatic No other complications
Sonnery‐Cottet et al. [101]	2014			TT + single‐stage	≥2nd revision ≥12° MA	8°−10° MA	After 31.6 months (mean, range, 23–45): No graft failure Good to excellent PROMs No complications
Vivacqua et al. [106]	2023	23		19 ST, 4 TT, 16 single‐stage, 7 two‐stage	First revision ≥12° PAA	n.a.	After 26.7 months (mean, range, 6–84): Three graft failures (13.0%, thereof two traumatic) Moderate PROMs One complex regional pain syndrome Two manipulation under anaesthesia
Weiler et al. [113]	2022	58		48 IT, 10 ST	4× Primary 23× first, 16× second, 11× third and 4× fourth revision individual decision	n.a.	No clinical results presented
Song et al. [99]	2020	18		IT + single‐stage	Primary non‐contact ACL insufficiency + chronic situation (>6 months from injury) + excessive static anterior translation + medial meniscus posterior horn lesions >13° MA	n.a. (Wedge height 8–10 mm)	After 33.2 months (median, range, 25–44): No graft failure SSD 13.0–1.6 mm Excellent PROMs 100% return to preinjury Level of activity No complications

Abbreviations: AA, anatomical axis; ACL, anterior cruciate ligament; FU, follow‐up; IT, infratuberositary; LoE, level of evidence; MA, mechanical axis; n.a., not applicable; PAA, proximal anatomical axis; PROMs, patient‐reported outcome measures; SSD, side‐to‐side difference; ST, supratuberositary; TT, transtuberositary.

In the opinion of the authors, no clear indication criteria can be defined according to the currently available literature. The recommendation to carry out or consider a correction from 12° is very common; however, using only a single threshold is surely an oversimplification of a complex problem for several reasons.

First, the often proposed threshold of 12° is often used without clearly referring to the measurement method used; and it has never been clinically evaluated in comparative studies. Further, the proposed 12° is not based on clinical data from patients who underwent these surgeries, but originates historically from studies that analysed failure rates after ACLR and then defined this value as the cut‐off in terms of a maximum mathematically defined threshold [34, 53, 87, 111]. Incorrectly, this suggests that a value of >12° would lead to an abrupt increase in the risk of ACL re‐rupture. But this is a misinterpretation of the actual conditions. Biomechanically, it has been shown that the force acting on the ACL increases approximately linearly with increasing PTS [1, 4, 43]. Further, large clinical studies showed an approximately linear relationship between higher PTS and the risk of ACL re‐rupture [26]. However, the clinical data on this is inconsistent, as Pineda et al. reported on a nonlinear relationship with a disproportionate increase of the risk of a re‐rupture in a high‐risk cohort of primary ACLR in the range of higher PTS values [82]. Nevertheless, descriptive analyses of various patient collectives with recurrent instabilities also showed that there is not a sharp increase at a certain value, but rather a flowing transition [3, 34].

Therefore, it must be concluded that defining a single threshold value can neither be adequate nor sufficient to clearly define the indication for PTS‐changing osteotomy. This is in line with a recently published consensus statement from an international expert group where an agreement of 96% was found for the statemen: ‘There is no single PTS measurement that represents an absolute indication for surgical correction with ACW high tibial osteotomy in the setting of revision ACLR—multiple factors must be considered, including patient activity goals, reason for previous ACL graft failure, previous ACLR technique, coronal alignment, status of menisci and cartilage and specific physical examination findings, including hyperextension of the knee’ [107].

Second, a PTS of ≥12° according to the PAA is a generally common condition and was found to be present in more than 32% of ACL injuries, 35% of ACL revisions, 45% of multiple ACL revision cases—and even 20% in healthy controls [3, 112]. Based on the proportion of ‘pathological’ cases in these collectives, this would subsequently result in an enormous number of osteotomies, which is surely not adequate with regard to overall satisfying results in the majority of revision ACLR [9, 59, 75].

Third, an ACL (re‐)rupture is a complex multifactorial event with multiple other conditions and risk factors apart from the PTS [38, 84, 96, 120]. These factors play key roles in the strategy of all ACL revisions, even without considering the PTS. However, many of these additional factors and conditions are directly or indirectly related to the PTS. It is crucial to systematically evaluate and consider these when determining the strategy for ACL revision, and even more when deciding whether a bony correction is indicated. The following key points are essential to be considered when the indication for slope‐changing osteotomy is evaluated:

− High‐grade laxity and distinct ATT (especially with excessive static ATT) are associated with a high PTS [26, 29, 68, 100, 120].
− Chronic ACL insufficiency might lead to laxity with subsequent higher degrees of instability [50, 119].
− Meniscus lesions (esp. ramp and root tears) are associated with a higher PTS [5, 24, 52, 65, 70].
− Non‐contact injuries have been shown to be more associated with a high PTS than contact injuries [8].
− Inadequate trauma mechanisms might be a sign of chronic overload, potentially caused by a high PTS (according to the authors' experience).
− Repeated graft failures are highly correlated with a high PTS [3, 34].
− A technically incorrect ACLR might fail without the PTS being the major factor [120].
− Contralateral ACL injuries are an independent risk factor for further injuries, and in bilateral ACL injuries, a higher PTS has been reported [27].
− A short time period between reconstruction and failure has been reported to be associated with a high PTS [3, 34].
− The sports level has to be considered as there is not much data available with regard to return‐ to‐ sports and preinjury level [57, 75, 99]. No data is available on PTS changing osteotomy in professional athletes.
− Younger age is associated with higher rates of subsequent re‐injuries, reflecting the fact that with individual risk constellations these injuries happen early in life [11, 84, 88, 96, 116].
− No difference has been reported between males and females with regard to the radiologically measured PTS [112].
− A family history of ACL injuries might lead to an overall increased risk constellation [120].
− Hyperextension can increase after PTS‐decreasing osteotomy, but has been reported to be asymptomatic in the majority of cases [57].
− Recurrent instability in cases of previously performed LET or anterolateral reconstruction is associated with a higher PTS [26, 64].

Schuster et al. recently proposed the so‐called ‘Avalanche Concept’—a comprehensive approach of individual decision making—taking the individual factors as well as the PTS into account [92]. It is an adopted decision‐making framework which has been proposed and used in avalanche safety, where the risk of an avalanche occurring is determined by the evaluation of individual risk factors on the one side and the steepness of the terrain (= PTS) on the other side. This is shown in Figure 4. The presence and severity of individual conditions and risk factors (listed above) should be analysed and ultimately mapped to an increasing degree via the *x*‐axis. This axis is intentionally not scaled or specified more precisely, as the individual significance of the respective factors cannot be clearly stated yet because of a lack of clinical data. At this time, a more precise scaling would be an oversimplification, much like a clinical score would be. With increasing data, it might be possible to define this axis more precisely in the future. For the moment, it remains a conceptual approach. However, many risk factors and increasing severity of those lead to a point far on the right on the *x*‐axis. The PTS (ideally measured according to the MA) is plotted on the *y*‐axis. The point defined this way in this coordinate system then defines the strength of the recommendation for an additional bony correction by the background colour of the underlying heat map. The authors recommend considering a PTS‐decreasing osteotomy in revision cases from a PTS ≥ 10° according to the MA and necessarily taking all factors listed above into account. Different heat maps for revisions and primary situations have been proposed, as slope‐changing osteotomy might also play a role in primary cases with high or excessive slope and severe additional risk factors, as proposed by Song et al. [99]. Recently, Dejour et. al. advocated that PTS‐reducing osteotomy should be considered for selected cases in primary ACLR when PTS exceeds 12°–14° too [20].

**Figure 4:**
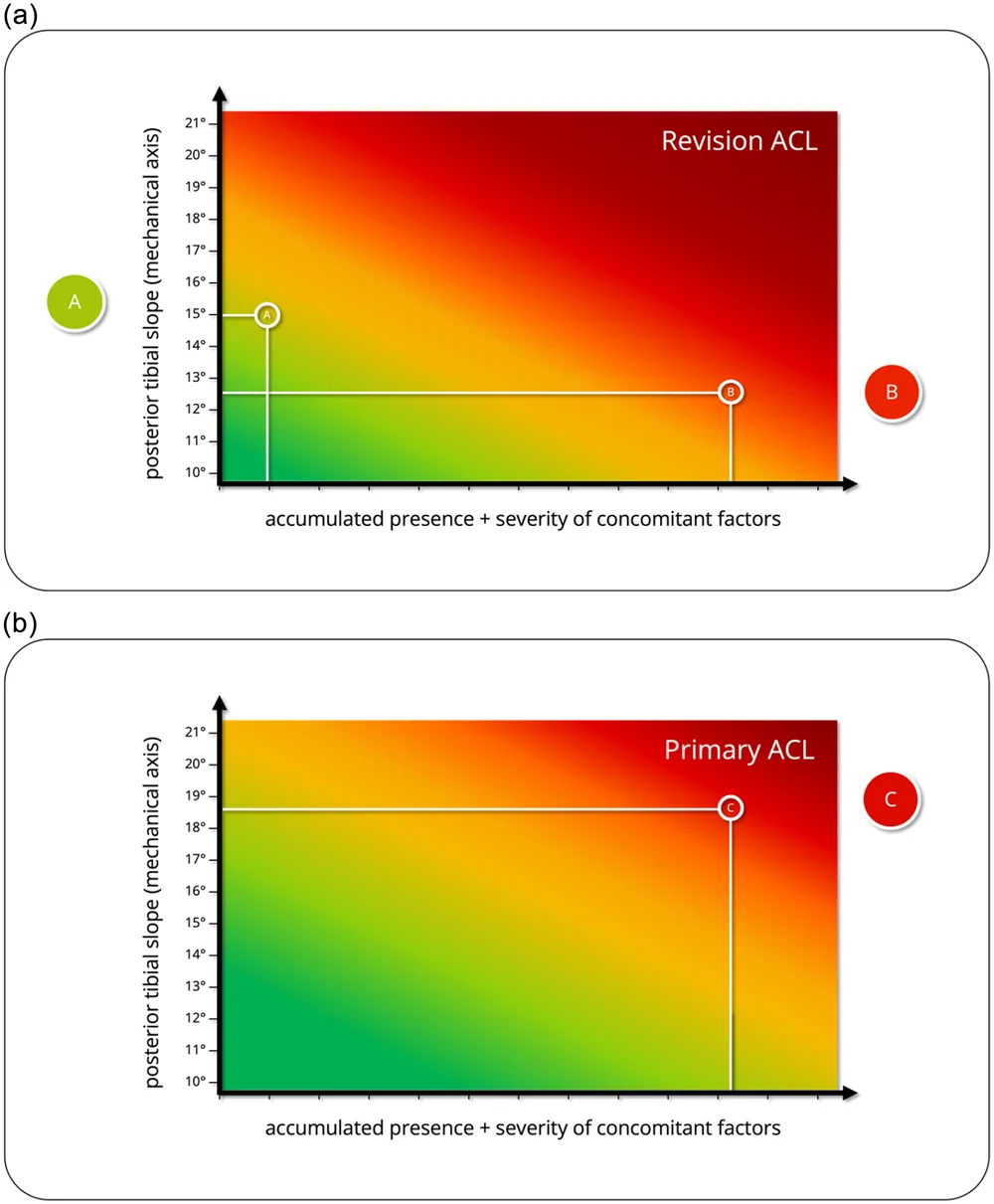
Heat maps for the indication of a posterior tibial slope (PTS) decreasing osteotomy for the different settings of revision (a) and primary cases (b). Patient‐specific factors are plotted on the *x*‐axis, and the radiologically measured PTS on the *y*‐axis. The point defined this way in this coordinate system defines the strength of the recommendation for PTS decreasing osteotomy by its underlying colour: dark red indicates a strong recommendation for PTS decreasing osteotomy, whereas green means no recommendation for osteotomy. The white circles represent different exemplary possible patient conditions (a, b): Patient A has a high PTS of around 15° (MA) but has no further risk factors. The heat map shows ‘green’. This patient might preferably be treated with ligament reconstruction only. Patient B has a moderately increased PTS of around 12.5° but has a number of severe concomitant factors, resulting in ‘red’, and thus resulting in a strong recommendation for concomitant osteotomy. Patient C has a primary ACL injury (b). With a number of severe concomitant risk factors and a high PTS of 18.5°, the heat map shows ‘dark orange’, and ACL reconstruction combined with a slope‐changing osteotomy might be considered and discussed in this case. (reprinted with permission, Schuster et al. [92]). ACL, anterior cruciate ligament; MA, mechanical axis.

A thorough assessment of the risk factors mentioned above, based on the current literature, is not yet possible. Therefore, it is important to clarify that this model and the used background colours should generally be understood more as a path for complex decision‐making rather than a model to clearly produce a yes or no answer, as scoring systems would do.

The current literature on ACW slope‐changing osteotomies is limited to a small number of Level 4 and Level 3 studies, with all being either retrospective analysis or prospective case series without control groups [2, 18, 19, 57, 86, 99, 101, 106, 113]. Table 1 provides a detailed overview of the most important studies in this field. No comparative studies are yet available regarding ACLR versus ACLR plus osteotomy, and no studies evaluated isolated slope‐changing osteotomy without ACLR.

In general, all these studies show promising results in a medium‐term FU, with good to excellent stability, significant improvements in patient‐reported outcome measures (PROMs), no or very few re‐ruptures and very low complication rates (Table 1).

**Recommendation for indication and decision making for PTS decreasing osteotomies**

– The authors recommend an individual approach taking into account specific patient characteristics, including injury‐associated risk factors in their respective manifestations on the one hand and the radiologically measured PTS on the other hand (‘The Avalanche Concept’).
– No strict thresholds should be used for the decision whether an osteotomy should be performed or not.

## SURGICAL TECHNIQUE OF ACW SLOPE DECREASING OSTEOTOMIES

### Preoperative considerations and planning

A thorough understanding of the underlying deformity is required for surgical planning. In addition to lateral radiographs of the lower leg, a long‐leg standing radiograph is mandatory to determine additional varus and valgus deformities. If incidental pathologies, such as torsional deformities or patellofemoral malalignment, are suspected, additional measurements using MRI or CT might be indicated. If an additional varus or valgus deformity needs to be treated, other treatment methods such as multiplanar osteotomies or double‐level corrections should be considered, which has already been discussed before (‐> indication) [67, 91]. The following paragraph refers specifically to ACW.

In general, PTS decreasing osteotomies of the proximal tibia can be performed either proximally, transversely or below the tibial tuberosity (Figure 5). Preoperative planning of the wedge height in ACW osteotomies requires lateral radiographs as described before (Figure 6) [110]. A new tibial plateau line is defined from the joint axis point to form the desired angle with the MA. If the proximal entry point of the osteotomy on the proximal tibia is changed downwards at the same angle around the centre of rotation hinge point, the corresponding wedge height can be planned. Alternatively, tools can be used to measure the angle and determine the second osteotomy plane [110]. The ratio between gap measurement (mm) and angular measurements is lower in trans‐ or infratuberosity osteotomies [83].

**Figure 5:**
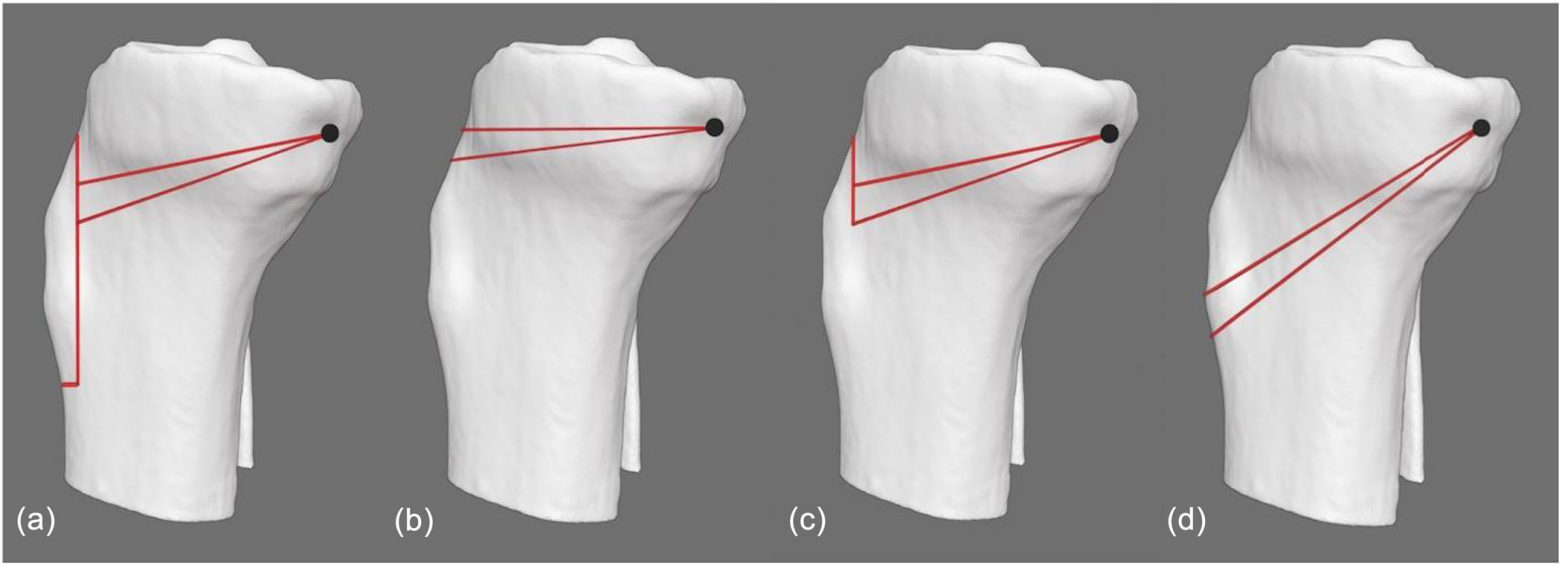
Surgical options for anterior closed wedge osteotomies: transtuberositary (a), supratuberositary (b), tubercle‐sparing retro‐tuberositary (c) and infratuberositary (d) approach.

**Figure 6:**
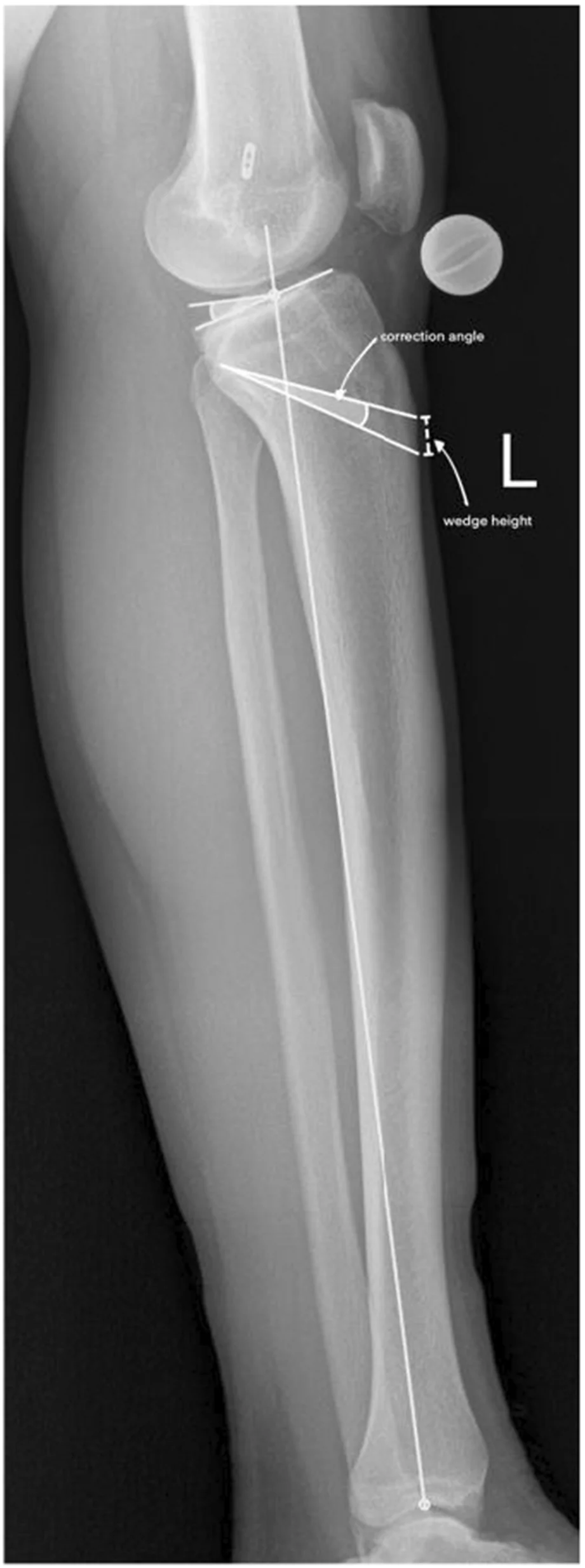
Planning of an infratuberositary anterior tibial closing wedge osteotomy on a lateral radiograph; the hinge point is determined at the lower half of the tibial footprint of the PCL (‘champagne glass drop’), the correction angle and/or the wedge height can be measured (for height measurement in millimetres (mm), a reference sphere is recommended). PCL, posterior cruciate ligament.

The optimal target range for postoperative PTS has not yet been clinically evaluated. Available studies have chosen different target values for PTS correction, ranging from 0°–5° to 8°–10° (Table 1), but these have never been compared with each other [2, 18, 19, 86, 101]. Dan et al. measured SATT before and after PTS decreasing osteotomy and reported a clear correlation to the postoperative PTS [12]. They defined a range of 0–5 mm of static ATT as their goal to be achieved and found this in cases with a postoperative PTS of 4°–6° (all according to the PAA). Although no additional subjective outcome scores or clinical data are reported in this study, it remains the only available investigation on this topic and currently represents, in the opinion of the authors, the commonly accepted target range for postoperative PTS.

### Surgical approaches

All described approaches for ACW osteotomies use the same hinge point in the area of the footprint of the posterior cruciate ligament (PCL) on the posterior tibia. No comparative studies are available for the different approaches, and they all have their specific advantages and disadvantages [94]. Dejour et al. described the supratuberositary (ST) approach in 1998, and recently this technique was re‐described [15, 31]. The osteotomy is completely proximal to the tuberosity. This technique is relatively well studied, as many case series have used it [18, 86]. One disadvantage may be that the remaining bone stock is limited for tibial ACL tunnel placement [94]. However, due to a very high primary stability, fixation with staples is usually sufficient, and therefore there is usually enough space for the desired tunnel placement [18, 94]. Nevertheless, tibial tunnels might be rather short. The overall complication rates described are low [18, 86, 106]. It is technically more demanding than other approaches, needs more surgical exposure around the patellar tendon and the proximal MCL, which need to be protected [93].

A transtuberositary (TT) approach was reported by several authors in one‐ or two‐stage procedures [2, 101]. The tuberosity is detached, the osteotomy is performed behind it, and after closure of the osteotomy, the tuberosity is reattached and used as a bio‐plate. Nevertheless, additional fixation with staples, screws or plates seems necessary [94]. ACL tunnel placement is less challenging than in ST approaches, but might still be limited or interfere with the fixation [94, 101]. However, the major disadvantage of this technique is the mandatory osteotomy of the tibial tubercle itself, which bears the risk of major complications like non‐union or fracture and does not allow accelerated rehabilitation [47, 56, 101].

The original authors of the technique later adapted their procedure and propagated a tubercle‐sparing osteotomy [32, 101]. In terms of its localization, this is a transtuberosity osteotomy, as it is located behind the tibial tubercle. This technique addresses the challenges associated with tuberosity osteotomies, as the integrity of the tibial tuberosity remains untouched and enables a gradual progression to full weightbearing. Nevertheless, it might bear the risk of a fracture of the tuberosity and is technically demanding.

In TT approaches, the patella height can at least theoretically be adjusted and maintained [2, 94, 101]. With ST or retro‐tubercle approaches, however, patella height is altered in roughly one third of cases, particularly when using indices that reference patellar height relative to the tibia [18, 32]. These are essentially minor changes and occur in both directions [32]. The clinical significance of these is unclear, and in short to medium‐term FU, no resulting disadvantages have been reported in small case series [57, 86].

The infratuberositary (IT) approach leaves the tuberosity completely intact, and no exposure or preparation is necessary around the patellar tendon [36, 71, 72, 93, 99]. It is a relatively simple technique that requires only minimal exposure, making minimally invasive surgery feasible [22, 76, 77, 93]. This approach allows sufficient space for a tibial tunnel, and changes in patellar height were described to be very rare [62, 93, 94, 99]. The primary stability is not as high as in ST approaches; therefore, fixation with angular‐stable implants is recommended [93, 99, 113]. For this reason and also because the cut is slightly more metaphyseal, compared to the other approaches, it has been discussed whether this approach might be associated more frequently with problems regarding bone healing [94]. However, this has recently been clearly contradicted, and an extremely low complication rate of only 0.6% for impaired bone healing had been reported in a large series with return to full weight bearing after 2–4 weeks [93].

### Recommended surgical technique for PTS decreasing osteotomies

The IT approach is recommended because of its major advantages, namely the easy and safe exposure, sufficient bone stock for tibial tunnel placement, possibility of early weight bearing and low complication rate in the absence of major disadvantages (Figure 7).

**Figure 7:**
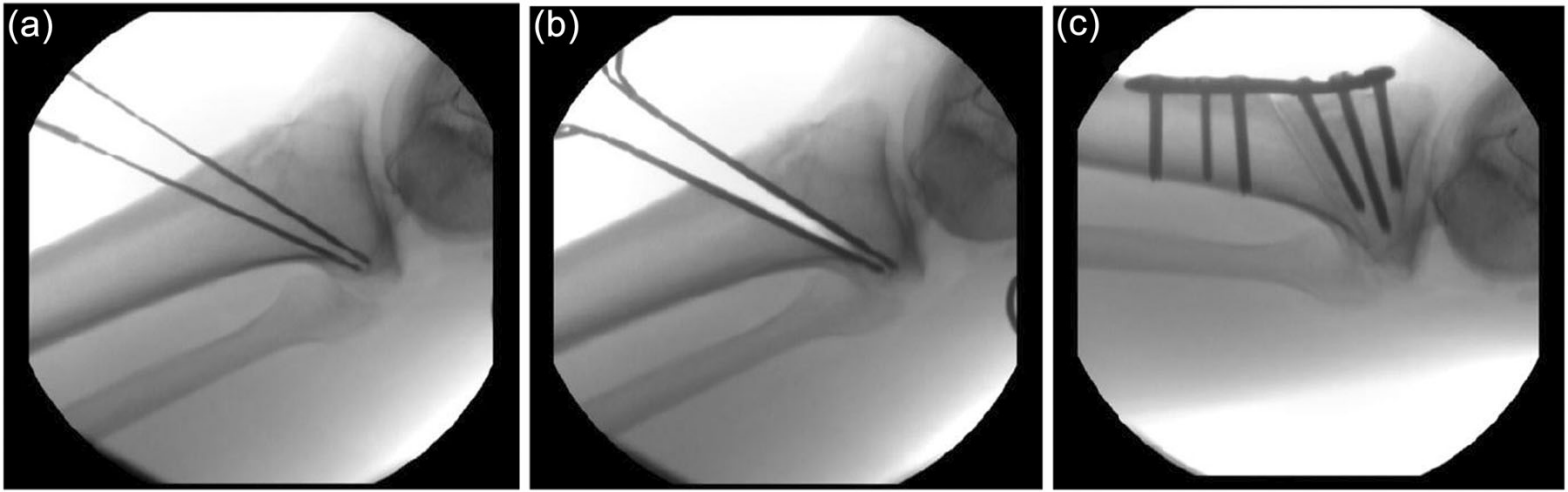
Surgical technique of an anterior tibial closing wedge osteotomy; radiographs are showing the proximal part of the tibia in a straight lateral view (joint lines aligned, posterior tibial condyles exactly superimposed); (a) Placement of the guiding K‐Wires. (b) Bone wedge extracted from osteotomy. (c) Closed osteotomy with angle stable plate fixation.

### Surgical technique as approached by the authors

A medial incision is made obliquely or parallel to the anterior tibial crest, depending on the available surgical approaches or the surgeon's preference. Specially developed implants for ACW osteotomies enable a minimally invasive anterior approach. A subperiosteal detachment with soft tissue protection is made at the level of the osteotomy. The anterior compartment is accessed via an incision in the fascia, and the very proximal part of the tibialis anterior muscle is then slightly retracted. On the medial side, the medial collateral ligament (MCL) is exposed, minimally elevated subperiosteally and protected. A small radiolucent retractor is recommended for protection of the MCL.

The osteotomy is marked with K‐wires, which are placed under fluoroscopic control. The tibial condyles should be exactly superimposed, and the joint gap should be clearly visible on lateral radiographs [93]. The hinge point is determined at the tibial footprint of the PCL (‘champagne glass drop’ on the tibia). It is recommended to aim at the lower half of the footprint, as the resulting hinge axis then corresponds more to a straight line compared to a more proximal located hinge. The distal osteotomy plane should pass just proximal to the tibiofibular joint, maintaining its integrity. Either two or four K‐wires are used. With two K‐wires, the saw blade must be visible completely as a straight line in the lateral radiograph for both cuts to ensure two parallel cuts. When four K‐wires are used, the proximal and distal two need to be perfectly superimposed on the lateral radiograph.

For a stable hinge, the final hinge axis should be parallel to the dorsal edge of the tibia. The wedge must be removed completely to avoid a hinge fracture. Some drilling or careful sawing (under radiographic control) into the dorsal cortex can facilitate closure of the osteotomy. The osteotomy should be closed smoothly. After complete closure, the plate is positioned and fixed proximally; an eccentric drill hole in a combi‐hole enables compression of the osteotomy. Depending on the implant design, an additional compression screw can be placed perpendicular to the osteotomy. Usually, no drains are necessary.

If no additional procedures are performed, full weight bearing as tolerated according to the implant's biomechanical properties is possible. Generally, two to four weeks of partial weight bearing is recommended. A brace is usually not necessary; however, to avoid distinct hyperextension in selected cases, a brace avoiding hyperextension in the first weeks might be helpful. Implant removal depends on individual circumstances (e.g., planned revision ACLR) and is usually possible after 3–4 months [93].

### Limitations

There are several limitations that have to be acknowledged. As a narrative review, a non‐systematic search methodology was applied, which might favour a selection bias. The underlying evidence lacks randomized controlled trials and comparative studies. Due to this situation, the conceptual approach of the Avalanche Concept has not yet been validated, but with current evidence it cannot and should not be used to provide an absolute yes‐or‐no‐decision. Further, the proposed optimal target PTS has not been clinically validated. In addition, no data on long‐term outcomes (>10 years) or on professional athletes are available. This lack of evidence needs to be addressed in future studies. Given the composition of the committee, a significant portion of the cited literature also originates from this research group, which might be a potential source of bias. However, the topic restricts high numbers, and the available evidence in this field originates from a limited number of specialized institutions. In addition, there are significant ethnic and regional differences in PTS and resulting treatment, but the available clinical data originates mainly from Europe and Asia. This needs to be considered.

## CONCLUSION

Recommendations for the management of increased PTS in ACL deficiency of the Osteotomy Committee of the German Knee Society:

1. Measuring the PTS on radiographs: Measuring should be performed on long lateral radiographs using the MA, with attention to a correct rotational alignment (exact superimposition of the posterior tibial condyles).
2. Indication for surgical correction of the PTS: Indication does not depend on a single fixed cut‐off value of the PTS. It should be based on an individual risk assessment (e.g., degree of ATT and instability, meniscal integrity, number and quality of previous ACLR, trauma mechanism: contact vs. non‐contact, contralateral injury).
3. Surgical technique to correct the PTS: For correction, different surgical techniques of ACWs have been reported without clear superiority of one technique. However, an IT approach is recommended, because it has been shown to be technically easy and safe.

## AUTHOR CONTRIBUTIONS

All authors contributed to the study conception, design and discussed the consensus topics. Material preparation, data collection and analysis were performed by Philipp Mayer. The first draft of the manuscript was written by Philipp Mayer, Julian Fürmetz and Philipp Schuster. All authors commented on previous versions of the manuscript. All authors read and approved the final manuscript.

## CONFLICT OF INTEREST STATEMENT

JF recieved financial support for a separate research project by Newclip. GB received speaker fees from Arthrex, Plasmaconcept, Enovis and is a paid Consultant for Arthrex. JH is a paid Consultant for Newclip. FI is a Consultant for Arthrex and Consultant for Enovis and also recieved a research Grant from Medacta. PS received speaker fees from Newclip Technics and Karl Storz SE and also received royalties from Newclip Technics. The other authors declare no conflicts of interest.

## ETHICS STATEMENT

The authors have nothing to report.

## Data Availability

Permission to republish Figure 4 was granted by the authors (Schuster et al.; senior author of the current publication). Since the article was published under an open access license, the copyright to the figure belongs to the authors themselves (https://link.springer.com/article/10.1007/s00402-025-06162-7). As this is a narrative review, there is no data that could be shared except for the literature, which is completely listed in the reference section.
